# Does a Vagal Response Indicate Cardiac Autonomic Modulation and Improve the Therapeutic Effect of Pulmonary Vein Isolation in Patients with Paroxysmal Atrial Fibrillation? Insights from Cryoballoon Ablation

**DOI:** 10.3390/jcdd9050142

**Published:** 2022-05-02

**Authors:** Radoslaw M. Kiedrowicz, Maciej Wielusinski, Marcin Zakrzewski, Jaroslaw Kazmierczak

**Affiliations:** Cardiology Department, Pomeranian Medical University, Powstancow Wlkp. 72, 70-111 Szczecin, Poland; metiu.w@interia.pl (M.W.); mar.zakrzewski@wp.pl (M.Z.); kardio@pum.edu.pl (J.K.)

**Keywords:** atrial fibrillation, cardiac autonomic nervous system, cryoballoon ablation, ganglionated plexi, heart rate variability, vagal response

## Abstract

*Background:* The incidence and influence of vagal response (VR) observed during cryoballoon-based pulmonary vein isolation (CBA-based PVI) on the cardiac autonomic nervous system (CANS) and ablation outcomes in paroxysmal atrial fibrillation (PAF) remain unknown. *Methods:* 296 patients were treated with a 28 mm second-generation cryoballoon (Medtronic). A total of 74 patients without structural heart disease and concomitant diseases were chosen for a detailed CANS assessment with a heart rate variability (HRV) analysis. All patients were screened over a 2-year post-ablation period. *Results:* VR was detected in 30% of patients and included sinus arrest (64%) or severe sinus bradycardia (46%). The presence of VR was not related to PV ostial dimension, patient clinical characteristics or intraprocedural ablation details. CANS modulation, manifesting as increased median HR and decreased HRV parameters with intact sympatho-vagal balance occurred independently of VR presence or absence and sustained for at least 12 months following ablation. VR was not related with more intensive CANS modulation and did not translate into better ablation outcomes when compared to the non-VR group (74% vs. 71% at 12 months and 69% vs. 65% at 24 months respectively). *Conclusions:* VR is frequent during CBA-based PVI for PAF and unrelated to any additional clinical benefit.

## 1. Introduction

An interplay between left atrial (LA) ganglionic plexi (GP), a part of the intrinsic cardiac autonomic nervous system (CANS) and pulmonary veins (PVs) is considered to be an important mechanism related to the initiation and maintenance of atrial fibrillation (AF) [1]. RF-based PV isolation (PVI) has been reported with better ablation outcomes when augmented with additional GP ablation [2]. However, the benefit of GP ablation remains controversial [3]. Cryoballoon ablation (CBA) is an anatomically based approach which allows highly safe and efficacious PVI [4]. The injury and subsequent scar created by the cryoballoon frequently extends beyond the PV orifice in the acute and chronic post-ablation phase [5,6] creating a set of lesions that are near the LA-GP area causing inadvertent damage [6]. Many studies have shown that CBA-based PVI is related to CANS alteration [7,8,9,10,11,12,13]. A marked vagal response (VR), relatively commonly observed during CBA, is generally considered a marker for the CANS modification [7,8,9,10,11,14,15,16,17] and has been purported to increase ablation success [7,14,15,17], although with conflicting results [9,10]. However, only small cohort studies selectively evaluated the impact of VR on CANS modulation [7,9,10,11]. Moreover, within those studies, autonomic changes were appraised with various, sometimes insufficient criteria [18]. Finally, it is very likely that the recruitment of unselected patient cohorts [8,9,10,11,12,14,15,16,17] could have led to CANS assessment bias. Many factors beyond CBA affect the autonomic tone, especially the presence of structural heart disease or other concomitant diseases, pharmacotherapy, consumption of stimulants and persistent AF, often associated with LA remodelling. Therefore, we sought to evaluate the impact of VR observed during CBA-based PVI on CANS status with a widely accepted heart rate variability (HRV) analysis in individuals free of any concomitant diseases and drugs known to influence autonomic nervous system function in a large paroxysmal AF (PAF) cohort, along with VR impact on ablation outcomes.

## 2. Materials and Methods

### 2.1. Study Population 

Two hundred and ninety-six consecutive patients with PAF referred for PVI were included prospectively. The participants were recruited from 2015 to 2017. Individuals with a previous history of AF ablation procedures were excluded. The study complied with the Declaration of Helsinki, and all patients provided written informed consent. The study protocol was approved by a local institutional review board. Antiarrhythmic drugs were discontinued for at least 5 half-lives before the procedure. All patients were treated with a 28 mm second-generation cryoballoon (Arctic Front Advance^TM^, Medtronic, Minneapolis, MN, USA). The procedure was performed under conscious sedation without stopping oral anticoagulation. 

### 2.2. Pulmonary Veins Measurement

**Figure 1:**
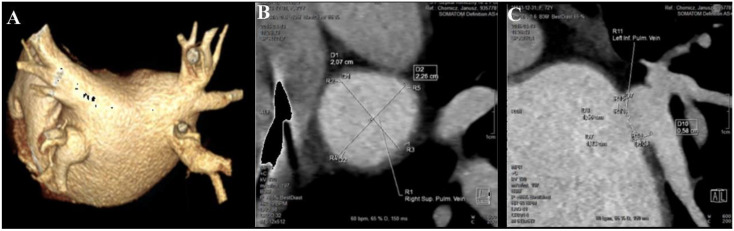
**Pulmonary vein anatomy addressed by a 64-row-multidetector computed tomography.** Three-dimensional left atrial reconstructed model in the postero-anterior view (panel **A**) and the example of pulmonary vein ostial maximum and minimum dimension analysis obtained from multiplanar reconstructed images using a dedicated caliper software: the right upper pulmonary vein in transverse cross-section (panel **B**) and the left inferior pulmonary vein in longitudinal cross-section (panel **C**).

The preprocedural anatomy of LA and PVs acquired with 64-row-multidetector computed tomography (CT) (Somatom Definition AS+, Siemens, Berlin, Germany) was available in 53% (156/296) of patients (Figure 1). The following indices were obtained from multiplanar reconstructed images for each of the PVs using dedicated calliper software [19]: PV maximum (PVmax) and minimum (PVmin) ostial diameter, PV elliptical area calculated as (PVmax/2) × (PVmin/2) × 3.14 and the ovality index calculated as PVmax/PVmin. 

### 2.3. Cryoballoon Ablation

A cryoballoon was introduced to the LA via a steerable sheath (Flexcath, Medtronic, Minneapolis, MN, USA) following a single transseptal puncture with a Swartz™ SL0 catheter (Abbot, Chicago, IL, USA). The balloon was advanced toward the PV ostium, either over a guidewire or an 8 polar-20 mm diameter circular catheter (Achieve^TM^, Medtronic, Minneapolis, MN, USA) and inflated. PV occlusion was documented by the injection of contrast. Optimal vessel occlusion was assumed when the PV showed complete contrast retention without any backflow to the atrium. PV anatomy was evaluated with an intraprocedural atriography where necessary. The freezing time was chosen between 180 and 240 s and left at the operator’s discretion, along with a decision to follow with a bonus- freeze cycle. The application was not stopped due to VR but resulted in temporary ventricular pacing, where necessary. The application was aborted, and the cryoballoon was repositioned in the case of ineffective cooling (inability to achieve a nadir temperature <−35 °C within 90 s of application) or when a nadir temperature decreased <−60 °C, to avoid excessive cooling. In cases where a real-time recording of PV potentials was available, a short time-to-isolation <60 s resulted in a single 180 s freeze cycle. CBA was always begun from the left upper PV (LUPV) followed by the left lower PV (LLPV). The presence of left common PV (LCPV) required an antral occlusion of the vein, validated by contrast injection. Otherwise, a sequential ablation approach targeting each PV branch was used. Ablation of the right-sided PVs was performed under continuous phrenic nerve pacing from the superior vena cava to avoid paralysis. The end of the procedure was decided when entrance block with the lack of capture within each ablated PV was achieved. This was verified either with a 20 pole-20 mm diameter circular mapping catheter (LASSO, Biosence-Webster, Irvine, CA, USA) or the Achieve catheter. To distinguish atrial far-field signals from PV potentials, coronary sinus pacing from different sites and/or superior vena cava pacing was performed where necessary. If residual PV potentials were evident, additional touch-up application was delivered. Vagal reactions were defined as sinus bradycardia <40 bpm, sinus arrest, atrioventricular block or hypotension registered at any time from the beginning of cryoapplication up to 1 min following balloon deflation preceded by a thawing period.

### 2.4. Heart Rate Variability Analysis

In order to eliminate all possible factors that might potentially affect the autonomic tone beyond CBA, only patients without structural heart disease and with no concomitant diseases (*n* = 74) were chosen for a detailed ANS assessment. A 24 h holter recording was taken on the day before ablation and 3, 6 and 12 months thereafter. No drugs beside oral anticoagulation were prescribed following ablation in this group. There was no need to use beta-blockers throughout follow-up. Caffeine, nicotine and alcohol were withheld throughout the 24 h testing period. The rationale for this was based on observations that caffeine has been noted to enhance both sympathetic and parasympathetic activity [20]; even second-hand exposure to nicotine decreases HRV by increasing sympathetic activity [21], and acute ethanol intake inhibits parasympathetic activity and results in the predominance of sympathetic nerve activity [22]. Holter electrocardiograms were recorded by a DMS 300-3A (DM Software, Tustin, CA, USA). HRV was analysed by a Cardioscan II system (DM Software). Artifacts, premature complexes and atrial runs were excluded from analysis. Records with abnormal beats, rhythms and noise that constituted >5% of all beats were repeated. Data analysis was performed according to widely accepted recommendations [18]. HRV parameters included mean heart rate (mHR), five time-domain variables ((1) standard deviation of all normal-to-normal (NN) intervals (SDNN), (2) a mean of the standard deviations of all NN intervals for all 5 min segments of the entire recording (SDNN index), (3) standard deviation of the averages of NN intervals in all 5 min segments of the entire recording (SDANN), (4) the square root of the mean sum of the squares of differences between adjacent NN intervals (rMSSD) and (5) number of pairs of adjacent NN intervals differing by more than 50 ms in the entire recording divided by the total number of all NN intervals (pNN50)) and three frequency domain variables ((1) spectral power at a low frequency range 0.04–0.15 Hz over the entire 24 h (LF), (2) spectral power at a high frequency range 0.15–0.4 Hz over entire 24 h (HF) and (3) low-frequency/high-frequency ratio (LF/HF)). All patients completed HRV evaluation at 3, 6 and 12 months post procedure. Records with AF were repeated where necessary.

### 2.5. Follow-Up Strategy

All patients were screened over a 2-year period. Outpatient visits were scheduled at 3, 6, 12, 18 and 24 months following ablation. At each visit, a detailed medical history was taken with emphasis on registered AF episodes or AF suggestive symptoms. 24 h Holter monitoring was performed at 3, 6, 12, 18 and 24 months after the ablation, and in any case, when arrhythmia recurrence was suspected. HRV analysis was not available beyond a 12-month post-ablation period. The primary study endpoint with respect to ablation efficacy was freedom from AF or other sustained (>30 s) atrial tachyarrhythmia after a single procedure beyond a 3-month blanking period. No antiarrhythmic drugs were allowed throughout the study.

### 2.6. Statistical Analysis

All continuous variables are expressed as median and interquartile ranges as they were not normally distributed. The categorical variables are presented as values and percentages. Comparisons between the groups were performed with either the Mann–Whitney U-test, the Wilcoxon test or the chi^2^ test, where appropriate. Survival curves were constructed using the Kaplan–Meier method and compared using the log-rank test. Statistical significance was accepted at *p* values < 0.05. Analysis was performed using Statistica software version 13.3 (StatSoft, Krakow, Poland).

## 3. Results

The baseline characteristics of the study cohort and CBA procedural details are shown in Table 1. Overall, 99% (1129/1137) PVs were successfully isolated. VR was detected in 30% of patients (89/296) and included sinus arrest (57/89, 64%) or severe sinus bradycardia (32/89, 46%). Atrioventricular block and hypotension were not observed. Among the HRV and CT scan group, VRs occurred with similar frequency to the overall patient cohort and was observed in 28% (21/74) and 30% (47/156) of patients, respectively. All VRs occurred during the thawing period or after balloon deflation, at a median time of 33 s (IQR 20–50 s) following freezing. VRs were observed during the LCPV isolation in 24% (11/47), LUPV isolation in 25% (62/249), LLPV isolation in 13% (32/249) and right upper PV (RUPV) isolation in 1% (3/249) of cases. Right lower PV (RLPV) isolation did not result in VR. Moreover in 20% of cases (18/89), VR was recorded during both LUPV and LLPV isolation, whereas in 3% (3/89) during LUPV, LLPV and RUPV isolation. The presence of VR was not related to age, sex, concomitant diseases, LA diameter, PV ostial diameters, cryoapplication time or minimal achieved temperature (Table 1).

Changes in HRV parameters from pre-ablation to post-ablation among the VR and non-VR group are shown in Table 2.

In both groups all HRV parameters with the exception of LF/HF ratio were significantly different following ablation and maintained up to 12 months. The comparison of pre- and post-ablation HRV parameters between the VR and non-VR group showed no significant difference.

**Figure 2:**
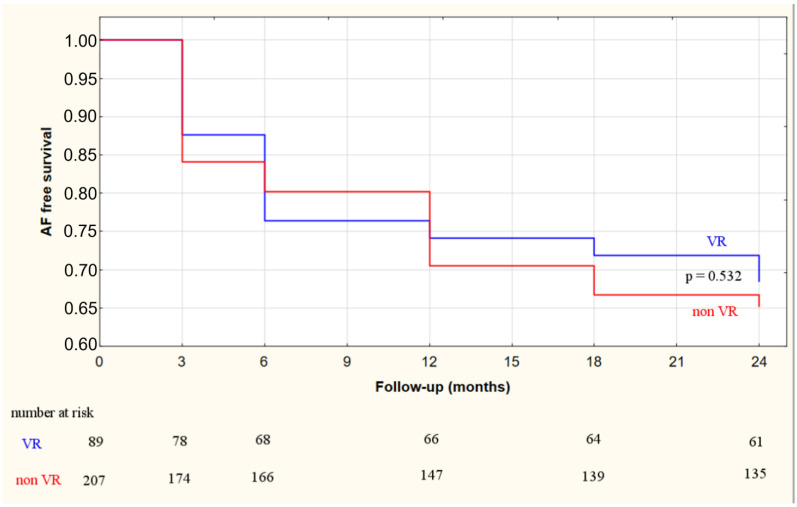
Atrial fibrillation free survival rate following cryoballoon-based pulmonary vein isolation beyond a 3-month blanking period across vagal response (VR) and non-vagal response (non-VR) subgroups.

The overall AF free survival rate beyond a 3-month blanking period was 72% (213/296) at 12 months following ablation and decreased to 66% (196/296) at 24 months. No significant difference of ablation efficacy between the VR and non-VR group was noted: 74% (66/89) vs. 71% (147/207) at 12 months (*p* = 0.39) and 69% (61/89) vs. 65% (135/207) at 24 months (*p* = 0.53), respectively (Figure 2).

Of the HRV population, 15 patients had a recurrence of arrhythmia. The AF free survival rate at 12 months follow-up for this group was 86% (64/74), decreasing to 80% (59/74) at 24 months. Changes in HRV parameters from baseline to post-ablation between the group who experienced recurrence and the non-recurrence group are shown in Table 3. All HRV parameters, with the exception of LF/HF ratio were found to be significantly different following ablation and continued up to 12 months in both groups. There was no difference between the group who experienced recurrence and the non-recurrence group when pre- and post-ablation HRV parameters were compared.

## 4. Discussion

The present study focused on the evaluation of VR incidence during CBA-based PVI among a large PAF cohort, along with its impact on CANS modulation and ablation outcomes as this is yet to be thoroughly investigated.

### 4.1. Incidence of VR during CBA-Based PVI

Within this study, it was found that VRs were frequent and presented exclusively as sinus arrest or bradycardia. In the other studies, VRs incidence were reported between 24–67% when PVI was started from left-sided PVs or an ablation sequence was not reported [7,8,9,10,11,14,15,16,17]. It seems that the discrepancy between the studies might be related to the relatively small number of patients recruited and, including persistent AF cases [11,14,15,16,17], which is often related to LA remodelling and subsequent CANS alteration [1,2]. Moreover, in one study VR was also considered when it occurred during balloon inflation or catheter manipulation [10]. When PVI was started from the right-sided PVs, VR incidence was reported to have decreased to 2–8% [8,10,16]. It was observed that the most common type of VR, that is, sinus bradycardia or arrest, was inhibited by initial RUPV isolation. This is explained by the fact that the ablation of RUPV impairs the efferent vagal neuron from anterior right GP (ARGP) that serves as the integration centre to modulate sinus rhythm. This is possible because the sinoatrial node is modulated by following pathway: superior left GP (SLGP) → ARGP → Sinus Node. Conversely, the incidence of AV block does not depend on the ablation sequence. The AV node is modulated by two pathways: SLGP → ARGP → inferior right GP (IRGP) → AV node and SLGP → IRGP → AV node, where IRGP is the integration centre for AV node modulation [16,23]. This can explain why VR was frequent during the presented study and limited to sinus arrest or bradycardia. Moreover, it was observed that VR presence was not predictable from PV anatomy, the clinical characteristics of our patients, or our intraprocedural ablation details. We can speculate that this phenomenon indicates that CBA-based PVI often results in wide antral tissue damage, especially in cases where a balloon was too large for one of the veins, and therefore GP activation.

### 4.2. Influence of CBA-Based PVI on CANS

The present study assessed the influence of CBA on autonomic balance with a widely accepted method, HRV analysis calculated over 24 h ECG recording [18]. This confirmed long-lasting (up to 12 months) CANS alteration, independently identified from the presence of VR. Moreover, VR is not related to more intensive autonomic modulation.

Several studies assessed the influence of CBA-based PVI on CANS [7,8,9,10,11,12,13], however with different, and not always sufficient surrogates [18], such as selected time-domain HRV variables [9], a decrease in the cycle length in a 3 min resting 12-lead ECG [10], difference in HR and systolic BP [7], selected frequency-domain HRV variables assessed intraprocedurally over a 2-min interval [8], selected time-domain and frequency-domain HRV analysis over a 3 min electrocardiogram recording [12] or finally HRV analysis over a 24 h ECG recording [11,13]. All of these methods showed that CBA-based PVI is related to CANS alteration. In some studies, these changes were transient, normalizing within 12 months following ablation [9,10,13], while long-lasting in others [11,12]. Small cohort studies which evaluated the impact of VR on CANS modulation [7,9,10,11] revealed that changes in the autonomic tone were independently noted from the presence of VR. The intensity of the CANS modification following ablation was similarly observed in both groups [11] or higher in the VR group [7]. The large general PAF cohort and detailed patient selection for 24 h HRV analysis in our study seems to be a major reason for the different results. The underlying mechanism of VR observed during ablation is not fully understood. In this study, detailed HRV analysis showed increased mHR along with decreased SDNN and derivates, RMSSD, pNN50 and HF, which are often considered to reflect altered parasympathetic activity [18]. However, LF/HF ratio that matches the sympatho-vagal balance [18] remained intact. These potentially conflicting results can be explained by the fact that actually both parasympathetic and sympathetic activities contribute to all HRV variables with a smaller proportion produced by unspecified factors [18]. Moreover, LA GP neuronal architecture is very complex, so it seems nearly impossible to selectively alter cardiac parasympathetic pathways. We do believe that GPs modification during CBA-based PVI involves both parts of CANS and the sympatho-vagal balance remains unchanged. Therefore, we can speculate that VR is related to transient vagal activation or indicates partial vagal denervation of GP.

### 4.3. Influence of VR on Ablation Outcomes

Several studies have suggested that GP ablation, as an adjunctive strategy to RF-based PVI, might be related to better clinical outcomes [2]. CBA-based PVI creates a lesion set which involves LA-PV junctions and frequently causes inadvertent GPs damage [6] which may potentially increase ablation success. Assuming that VRs during PVI indicate CANS modification, it would not be surprising if this influenced the post-ablation AF recurrence rate. There are conflicting results with regards to the association between intra-procedural VR and ablation results. VRs predicted better clinical outcomes in some studies [7,14,15,17]. However, in the others, VR did not show a favourable effect [9,10]. It was postulated that VRs may not be able to indicate complete autonomic denervation, or reinnervation of CANS may occur during the follow-up period. Moreover, the partial denervation of the autonomic nervous system may increase the incidence of AF [16]. Discrepancies between the studies might be related to the relatively small number of patients recruited and/or mixed AF populations.

This study showed that VRs do not translate into better ablation outcomes. This can be clearly explained by the fact that CANS modulation occurs independently from the presence or absence of VR, and VR is not related to more intensive CANS modulation.

### 4.4. Influence of CANS Alteration on Ablation Outcomes

It was found that CANS modification was a common phenomenon during CBA-based PVI but did not predict AF free survival. It could be hypothesized that GPs were only partially denervated during ablation, which was insufficient to fully alter autonomic tone. However, it seems that the results could have been biased due to particular patient selection for HRV analysis. The selected patients did not have structural heart disease or any concomitant disease beside AF. We can speculate that the majority of those individuals were free of atrial cardiomyopathy and abnormal substrate except for the PVs, which has been confirmed to improve AF ablation outcomes [24]. Therefore, PVI was distinctly more effective compared to the other group, probably suffering from more severe forms of disease. In this kind of patient cohort, it is not straightforward to demonstrate a clear relationship between GP ablation and more effective clinical outcomes.

### 4.5. Study Limitations

(1) Even though hypotension was considered as the indicative VR phenomenon, it was not observed in this study. It seems that the detection of BP drop is readily noted during continuous, invasive BP monitoring, which is usually not employed when the ablation procedure is performed under conscious sedation, as is the case at our centre. This might have potentially altered the VR incidence and dataset interpretation. However, in the majority of previous studies, hypotension was a very rare VR indicating phenomenon. (2) The overall ablation success rate clearly depends on reliable AF recurrence detection. Intermittent rhythm monitoring modalities used in the study were short-term and discontinuous. It cannot be ruled out that the use of long-term and/or continuous ECG monitoring might have potentially decreased the ablation success rate if it had been applied.

## 5. Conclusions

VR is frequent during CBA when PVI is initiated from left-sided PVs. It is unrelated to any additional clinical benefit because it does not translate to more intensive CANS modulation and does not improve ablation outcomes. It seems logical to avoid VR during CBA by starting PVI from right-sided PVs as recently reported. However, if phrenic nerve paralysis occurs, it can interfere with completion of the ablation process. Because VR is limited to sinus node suppression, it can be easily solved by atrial pacing. Therefore, we suggest starting PVI from left-sided PVs with atrial backup pacing.

## Figures and Tables

**Table 1:** Baseline characteristics of the study cohort (*n* = 296) and cryoballoon ablation procedural details.

	Entire Group	VR (+)	VR (−)	*p*
	*n* = 296	*n* = 89	*n* = 207	
Age, years	60 (54–64)	59 (55–64)	60 (54–65)	0.6
Females, *n* (%)	118 (40%)	33 (37%)	85 (41%)	0.3
Hypertension, *n* (%)	195 (66%)	58 (65%)	137 (66%)	0.7
Chronic coronary syndrome, *n* (%)	50 (17%)	14 (16%)	36 (17%)	0.8
Heart failure, *n* (%)	6 (2%)	1 (1%)	5 (2%)	0.5
eGFR <60 mL/min/1.73 m^2^, *n* (%)	7 (2%)	2 (2%)	5 (2%)	0.6
Diabetes, *n* (%)	41 (14%)	13 (15%)	28 (14%)	0.2
Left ventricular ejection fraction, %	60 (55–65)	60 (55–65)	60 (55–65)	0.9
Left atrial antero-posterior diameter, mm	43 (39–47)	42 (39–46)	43 (39–47)	0.9
CHA2DS2-VASc score	2 (1–3)	2 (1–3)	2 (1–3)	0.9
	6 (2%)	2 (2%)	4 (2%)	0.8
Prior history of ischemic stroke/TIA LCPV, *n*	47(16%)	12 (13%)	35 (17%)	0.2
LCPV ostial area, mm^2^	732 (436–800)	737 (431–791)	741 (431–811)	0.2
LCPV ostial ovality index	1.6 (1.4–1.7)	1.6 (1.3–1.6)	1.6 (1.4–1.8)	0.8
LUPV ostial area, mm^2^	235 (185–296)	216 (176–259)	228 (172–299)	0.3
LUPV ostial ovality index	1.5 (1.2–1.7)	1.6 (1.3–1.9)	1.5 (1.3–1.7)	0.9
LLPV ostial area, mm^2^	173 (149–214)	184 (162–199)	175 (148–217)	0.2
LLPV ostial ovality index	1.4 (1.3–1.7)	1.4 (1.3–1.7)	1.4 (1.3–1.7)	0.9
RUPV ostial area, mm^2^	297(227–361)	297(214–339)	292 (221–353)	0.7
RUPV ostial ovality index	1.3 (1.1–1.4)	1.3 (1.1–1.5)	1.3 (1.1–1.4)	0.8
RLPV ostial area, mm^2^	235 (179–297)	234 (187–293)	227 (177–299)	0.6
RLPV ostial ovality index	1.2 (1.1–1.3)	1.2 (1.1–1.3)	1.2 (1.0–1.3)	0.9
Number of applications, *n*				
LUPV	2 (1–2)	2 (2–2)	2 (1–2)	0.8
LCPV	2 (1.5–2)	2 (2–2)	2 (1–3)	0.9
LLPV	2 (2–2)	2 (2–2)	2 (2–2)	0.9
RUPV	2 (1–2)	2 (1–2)	2 (1–2)	0.9
RLPV	2 (1–2)	2 (2–2)	2 (1–2)	0.8
Freeze duration, s				
LUPV	480 (240–480)	480 (240–480)	480 (240–480)	0.9
LCPV	480 (340–480)	480 (480–480)	480 (240–480)	0.7
LLPV	480 (300–480)	480 (270–480)	480 (480–480)	0.7
RUPV	480 (240–480)	480 (240–480)	480 (240–480)	0.9
RLPV	480 (240–480)	480 (330–480)	480 (240–480)	0.6
Nadir temperature, °C				
LUPV	−48 (−53;−44)	−48 (−53;−43)	−48 (−53;−44)	0.8
LCPV	−50 (−54; −43)	−51 (−54; −43)	−50 (−54; −43)	0.6
LLPV	−43 (−48;−40)	−44 (−47;−41)	−43 (−48;−40)	0.7
RUPV	−48 (−53;−43)	−50 (−54;−45)	−48 (−53;−43)	0.3
RLPV	−44 (−51;−38)	−43 (−50;−38)	−44 (−51;−39)	0.5

VR(+), vagal response subgroup; VR(−), non-vagal response subgroup; LCPV, left common pulmonary vein; LUPV, left upper pulmonary vein; LLPV, left lower pulmonary vein; RUPV, right upper pulmonary vein; RLPV, right lower pulmonary vein.

**Table 2:** Changes in HRV parameters following ablation among the vagal (*n* = 21) and non-vagal response (*n* = 53) groups.

		Preablation	3 Months Post-Ablation	*p* Value vs. Preablation	6 Months Post-Ablation	*p* Value vs. Preablation	12 Months Post-Ablation	*p* Value vs. Preablation
**mHR, beats per minute**	VR(+)	70 (66–76)	81 (73–89)	<0.001	83 (73–90)	<0.001	78 (70–88)	<0.001
	VR (−)	70 (65–78)	78 (68–90)	<0.001	76 (68–84)	<0.001	76 (70–82)	<0.001
**SDNN, ms**	VR(+)	123 (108–190)	96 (70–101)	<0.001	102 (75–115)	<0.001	106 (89–140)	<0.001
	VR (−)	121 (102–155)	99 (74–112)	<0.001	108 (88–123)	<0.001	110 (90–130)	<0.001
**SDNN index, ms**	VR(+)	54 (44–93)	36 (24–69)	<0.001	38 (26–71)	<0.001	42 (29–78)	<0.001
	VR (−)	57 (45–102)	33 (24–71)	<0.001	37 (28–76)	<0.001	40 (30–75)	<0.001
**SDANN, ms**	VR(+)	115 (87–169)	93 (61–117)	<0.001	95 (65–121)	<0.001	104 (71–133)	<0.001
	VR (−)	103 (77–145)	78 (52–98)	<0.001	82 (63–108)	<0.001	89 (65–114)	<0.001
**rMSSD, ms**	VR(+)	51 (32–78)	30 (26–50)	<0.001	32 (28–48)	<0.001	34 (28–45)	<0.001
	VR (−)	39 (28–66)	28 (24–47)	<0.001	33 (23–42)	<0.001	33 (23–43)	<0.001
**pNN50, %**	VR(+)	19 (6–28)	4 (1–7)	<0.001	4 (1–6)	<0.001	5 (2–8)	<0.001
	VR (−)	13 (4–22)	3 (1–6)	<0.001	4 (1–6)	<0.001	4 (1–8)	<0.001
**LF, ms^2^**	VR(+)	599 (380–947)	377 (294–587)	<0.001	385 (290–592)	<0.001	417 (301–645)	<0.001
	VR (−)	593 (368–921)	385 (291–634)	<0.001	390 (292–674)	<0.001	402 (297–612)	<0.001
**HF, ms^2^**	VR(+)	203 (115–589)	144 (67–345)	<0.001	159 (74–360)	<0.001	167 (98–411)	<0.001
	VR (−)	187 (103–622)	147 (87–415)	<0.001	156 (93–420)	<0.001	164 (101–425)	<0.001
**LF/HF**	VR(+)	2.2 (1.5–4.1)	2.4 (1.5–4.5)	0.12	2.7 (1.7–4.4)	0.34	2.8 (1.8–4.3)	0.45
	VR (−)	2.3 (1.8–4.2)	2.5 (1.7–4.4)	0.2	2.6 (1.9–4.2)	0.45	2.6 (1.8–4.2)	0.51

VR(+), vagal response subgroup; VR(−), non-vagal response subgroup; mHR, mean heart rate; SDNN, standard deviation of all normal-to-normal (NN) intervals; SDNN index, standard deviations mean of all NN intervals; SDANN, standard deviation of the averages of NN intervals; rMSSD, the square root of the mean sum of the squares of differences between adjacent NN intervals; pNN50, number of pairs of adjacent NN intervals differing by more than 50 ms divided by the total number of all NN intervals; LF, spectral power in the low frequency range; HF, spectral power in the high frequency rang.

**Table 3:** Changes in HRV parameters following ablation among the recurrence (*n* = 15) and non-recurrence (*n* = 59) groups.

		Preablation	3 Months Post-Ablation	*p* Value vs. Preablation	6 Months Post-Ablation	*p* Value vs. Preablation	12 Months Post-Ablation	*p* Value vs. Preablation
**mHR, beats per minute**	recurrence	70 (66–76)	81 (73–89)	<0.001	83 (73–90)	<0.001	78 (70–88)	<0.001
	non-recurrence	70 (65–78)	78 (68–90)	<0.001	76 (68–84)	<0.001	76 (70–82)	<0.001
**SDNN, ms**	recurrence	123 (108–190)	96 (70–101)	<0.001	102 (75–115)	<0.001	106 (89–140)	<0.001
	non-recurrence	121 (102–155)	99 (74–112)	<0.001	108 (88–123)	<0.001	110 (90–130)	<0.001
**SDNN index, ms**	recurrence	54 (44–93)	36 (24–69)	<0.001	38 (26–71)	<0.001	42(29–78)	<0.001
	non-recurrence	57 (45–102)	33 (24–71)	<0.001	37 (28–76)	<0.001	40 (30–75)	<0.001
**SDANN, ms**	recurrence	115 (87–169)	93 (61–117)	<0.001	95 (65–121)	<0.001	104 (71–133)	<0.001
	non-recurrence	103 (77–145)	78 (52–98)	<0.001	82 (63–108)	<0.001	89 (65–114)	<0.001
**rMSSD, ms**	recurrence	51 (32–78)	30 (26–50)	<0.001	32 (28–48)	<0.001	34 (28–45)	<0.001
	non-recurrence	39 (28–66)	28 (24–47)	<0.001	33 (23–42)	<0.001	33 (23–43)	<0.001
**pNN50, %**	recurrence	19 (6–28)	4 (1–7)	<0.001	4 (1–6)	<0.001	5 (2–8)	<0.001
	non-recurrence	13 (4–22)	3 (1–6)	<0.001	4 (1–6)	<0.001	4 (1–8)	<0.001
**LF, ms^2^**	recurrence	599 (380–947)	377 (294–587)	<0.001	385 (290–592)	<0.001	417 (301–645)	<0.001
	non-recurrence	593 (368–921)	385 (291–634)	<0.001	390 (292–674)	<0.001	402 (297–612)	<0.001
**HF, ms^2^**	recurrence	203 (115–589)	144 (67–345)	<0.001	159 (74–360)	<0.001	167 (98–411)	<0.001
	non-recurrence	187 (103–622)	147 (87–415)	<0.001	156 (93–420)	<0.001	164 (101–425)	<0.001
**LF/HF**	recurrence	2.2 (1.5–4.1)	2.4 (1.5–4.5)	0.19	2.7 (1.7–4.4)	0.31	2.8 (1.8–4.3)	0.53
	non-recurrence	2.3 (1.8–4.2)	2.5 (1.7–4.4)	0.31	2.6 (1.9–4.2)	0.36	2.6 (1.8–4.2)	0.41

## Data Availability

The data presented in this study are available on request. The data are not publicly available due to ethical restrictions.
